# Geographic Access to High Capability Severe Acute Respiratory Failure Centers in the United States

**DOI:** 10.1371/journal.pone.0094057

**Published:** 2014-04-04

**Authors:** David J. Wallace, Derek C. Angus, Christopher W. Seymour, Donald M. Yealy, Brendan G. Carr, Kristen Kurland, Arthur Boujoukos, Jeremy M. Kahn

**Affiliations:** 1 Clinical Research, Investigation and Systems Modeling of Acute Illness (CRISMA) Center, Department of Critical Care Medicine, University of Pittsburgh School of Medicine, Pittsburgh, Pennsylvania, United States of America; 2 Department of Emergency Medicine, University of Pittsburgh School of Medicine, Pittsburgh, Pennsylvania, United States of America; 3 Department of Emergency Medicine, University of Pennsylvania Perelman School of Medicine, Philadelphia, Pennsylvania, United States of America; 4 Heinz College School of Public Policy and Management, Carnegie Mellon University, Pittsburgh, Pennsylvania, United States of America; D'or Institute of Research and Education, Brazil

## Abstract

**Objective:**

Optimal care of adults with severe acute respiratory failure requires specific resources and expertise. We sought to measure geographic access to these centers in the United States.

**Design:**

Cross-sectional analysis of geographic access to high capability severe acute respiratory failure centers in the United States. We defined high capability centers using two criteria: (1) provision of adult extracorporeal membrane oxygenation (ECMO), based on either 2008–2013 Extracorporeal Life Support Organization reporting or provision of ECMO to 2010 Medicare beneficiaries; or (2) high annual hospital mechanical ventilation volume, based 2010 Medicare claims.

**Setting:**

Nonfederal acute care hospitals in the United States.

**Measurements and Main Results:**

We defined geographic access as the percentage of the state, region and national population with either direct or hospital-transferred access within one or two hours by air or ground transport. Of 4,822 acute care hospitals, 148 hospitals met our ECMO criteria and 447 hospitals met our mechanical ventilation criteria. Geographic access varied substantially across states and regions in the United States, depending on center criteria. Without interhospital transfer, an estimated 58.5% of the national adult population had geographic access to hospitals performing ECMO and 79.0% had geographic access to hospitals performing a high annual volume of mechanical ventilation. With interhospital transfer and under ideal circumstances, an estimated 96.4% of the national adult population had geographic access to hospitals performing ECMO and 98.6% had geographic access to hospitals performing a high annual volume of mechanical ventilation. However, this degree of geographic access required substantial interhospital transfer of patients, including up to two hours by air.

**Conclusions:**

Geographic access to high capability severe acute respiratory failure centers varies widely across states and regions in the United States. Adequate referral center access in the case of disasters and pandemics will depend highly on local and regional care coordination across political boundaries.

## Introduction

An estimated 332,100 cases of severe respiratory from acute respiratory distress syndrome (ARDS) occur in the United States each year, resulting in approximately 133,500 deaths [1] as well as significant long-term morbidity [2], [3]. Treatment for ARDS and other forms of severe acute respiratory failure is resource intensive and requires specialized care for optimal patient outcomes [4]–[7]. This level of care is typically not available at all hospitals, suggesting that patient outcomes may be improved by directing more seriously ill patients to high capability centers [8].

There are no established hospital criteria for high capability centers for severe acute respiratory failure; however, candidate criteria include high mechanical ventilation hospital volumes or the ability to perform extracorporeal membrane oxygenation (ECMO). A volume-outcome relationship exists for mechanically ventilated medical patients, with higher annual hospital volumes associated with improved patient outcomes [9]. Likewise, patients treated at hospitals with ECMO capability have improved outcomes with severe ARDS [8] and a more than two-fold mortality benefit with influenza H1N1-associated ARDS [10].

Quantifying geographic access to hospitals with these capabilities has great public health importance. Determining the number, location, distribution and bed counts of these centers in the United States will help inform public health planning efforts. In addition, quantifying geographic access is a first step towards identifying regions with potentially strained resources, which has implications for routine critical care delivery as well as for pandemic event planning [11]. Severe respiratory illnesses caused by Middle East Respiratory Syndrome Coronavirus [12], avian influenza A [13] or other respiratory pathogens may place abrupt demands on regional intensive care resources underscoring the importance of a geographic access evaluation. Finally, this evaluation could inform a larger discussion regarding the value of regionalized intensive care for more broadly defined severe critical illness.

We sought to evaluate geographic access to high capability severe acute respiratory failure centers in the United States using two candidate criteria: (1) reporting adult ECMO cases to the Extracorporeal Life Support Organization (ELSO) or in Medicare discharge claims, and (2) reporting a high annual volume of mechanical ventilation in Medicare discharge claims from medical patients. In contrast to other time-sensitive medical conditions [14]–[16], severe ARDS can develop over days, often after hospital admission [17], [18]; therefore, we incorporated interhospital transfers into our geographic access calculations. We included long distance transits in our model as they are considered feasible and safe by ground [19] or air [8], [20] in this patient population. We determined geographic access to high capability centers using national census, air medical transport and street network databases.

## Methods

We performed a cross-sectional analysis of high capability severe acute respiratory failure center geographic access for the adult population based on previous resource allocation models [21], [22]. As many patients with severe ARDS are initially treated at community hospitals [8], we created a two-level population geographic access model using high capability center locations and referring hospital locations. We defined geographic access as the percentage of the adult population living within a one-hour driving radius of a high capability center, plus the percentage of the adult population living within a one-hour driving radius of hospitals that may refer patients to these centers. We used a one-hour driving radius to liberally estimate the hospital catchment population, based on prior studies that show 95% of emergency department patients live within 12 miles of the hospital [23], [24], but also expecting that patients with more severe symptoms will be willing to drive farther for emergency treatment [25]. For interhospital transport access, we performed separate analyses for both one-hour and two-hour transport intervals. We examined state-level, regional and national geographic access when patient transport between referring hospitals and high capability centers was conducted using ground or rotary air transportation.

### High Capability Severe Acute Respiratory Failure Center Criteria

We defined high capability severe acute respiratory failure centers using two hospital criteria: (1) provision of adult ECMO, based on either ELSO reporting or provision of ECMO to 2010 Medicare beneficiaries; or (2) high hospital mechanical ventilation volume, based 2010 Medicare claims. We developed these criteria based on a conceptual model of high capability severe acute respiratory failure centers that recognizes the established volume-outcome relationships in mechanical ventilation and the frequent use of ECMO in severe influenza [26].

We used two data sources to identify high capability centers performing adult ECMO for respiratory failure. First, we used the ELSO website to identify hospitals performing adult ECMO [27]. ELSO maintains an on-line list of active adult ECMO centers that have submitted cases in the past five years. We excluded adult cases reported from children's hospitals, to identify hospitals that provide routine care of adult patients. Second, we used the 2010 Medicare Provider Analysis and Review (MedPAR) file to identify hospitals reporting ECMO in administrative claims. MedPAR includes the final action claims of all hospitalized fee-for-service Medicare beneficiaries and is the only national source of hospital claims data. We analyzed claims for patients 18 and older from the 50 United States and the District of Columbia. We identified ECMO using the International Classification of Diseases, 9^th^ Revision, Clinical Modification (ICD-9-CM) procedure code 39.65 [28].

To identify high capability centers using high mechanical ventilation criteria, we also used the 2010 MedPAR file, identifying adult, non-surgical patients receiving mechanical ventilation using ICD-9-CM procedure codes 96.70, 96.71, 96.72 and a non-surgical diagnosis related group code [28]. We defined hospitals as having a high volume of mechanical ventilation if they reported more than 315 mechanical ventilation claims from adult Medicare patients in 2010. We used this threshold based on a prior volume-outcome study in medical patients receiving mechanical ventilation [9] and the age distribution of medical patients receiving mechanical ventilation in the United States [29]. The 315 threshold in Medicare estimates an all-payer hospital volume of approximately 600 mechanical ventilation cases per year, calculated using the proportion of medical patients in the United States who are aged 65 or older and are mechanically ventilated (52%) [29].

### Other Data Sources

We used the 2009 American Hospital Association (AHA) Annual Survey to characterize hospitals, summarize ICU bed counts and obtain geographic coordinates [30]. We linked reporting hospitals with the AHA Annual Survey using the hospital Medicare Provider Identification number. We used the 2012 Atlas and Database of Air Medical Services (ADAMS) to identify hospitals that routinely receive rotatory air transfers of patients [31]. We calculated the population aged 18 and older using block group data from the 2010 United States Census [32].

### Hospital Characteristics Analysis

We summarized hospital characteristics for each high capability center criteria and for all short term acute care hospitals in the United States. Variables of interested included the number of hospital beds, number of intensive care unit (ICU) beds, United States region, (Northeast, Midwest, South or West), urbanicity according to the metropolitan statistical area designation of the hospital ZIP code (division: more than 2.5 million persons; metropolitan: between fifty thousand and 2.5 million persons; micropolitan: ten to fifty thousand persons; or rural), teaching status according to each hospital's resident to bed ratio (non-teaching: 0; small teaching: >0 to 0.2; large teaching: >0.2) and ownership status (nonprofit, for profit or government).

### Ground Transport Analysis

To identify hospitals potentially referring patients by ground, we analyzed road network and speed limit data from the ArcGIS StreetMap database using ArcInfo 10.1 (ESRI Corporation; Redlands, California) and the Network Analyst extension. All adult short term acute care hospitals located within one- and two-hour driving radii of high capability centers were considered referring hospitals.

### Rotary Air Transport Analysis

We identified all high capability centers that routinely receive rotary air transports using the ADAMS database [31]. We then identified all adult short term acute care hospitals within a one hundred twenty (one-hour) and a two hundred forty (two-hour) mile geodesic radius of air-capable high capability centers. We used these distance based on the typical one- and two-hour flight characteristics of rotary aircraft reported to ADAMS.

### Population Geographic Access

We compared state-level, regional and national geographic access using each high capability center criteria. Our analysis included all adult United States residents, excluding those living in United States territories. We used the geometric center of each United States Census block group to summarize the population with high capability center geographic access. We did not include day of week or time of day in our calculations based on prior work that showed these variables to have a negligible effect, on average, on transport time estimates for emergency medical transports [33].

We created maps of ground and rotary air coverage using ArcGIS version 10.1 software (ESRI, Redlands, CA). We created two types of geographic access maps: continuous Albers equal area projections and isodemographic cartograms using the Gastner-Newman method of spatial transformation [34]. In the isodemographic projections, state geometry is distorted proportionally to the state population.

We analyzed data analysis using STATA 12.1 (StataCorp, College Station, TX). This research received human subjects review approval by the University of Pittsburgh.

## Results

In 2010, of 4,822 acute care hospitals, there were 498 (10.3%) high capability severe acute respiratory failure centers in the United States. We identified 148 hospitals meeting our ECMO criteria and 447 hospitals meeting our mechanical ventilation criteria. A minority of hospitals (n = 97/498, 19.5%) met our criteria for both ECMO and high annual volume of mechanical ventilation.

Both high capability center criteria identified hospitals with higher median numbers of hospital beds (501 for ECMO criteria and 489 for high volume mechanical ventilation criteria) and ICU beds (64 and 54, respectively) compared to all acute care hospitals in the United States (Table 1). The ECMO criteria identified a greater proportion of large teaching hospitals compared to the high volume mechanical ventilation criteria (49% and 33%, respectively). High capability centers were located predominantly in urban areas.

**Table 1:** High Capability Severe Acute Respiratory Failure Center Characteristics.

	ECMO Criteria^a^ (n = 148)	High Volume Mechanical Ventilation Criteria^b^ (n = 447)	All Acute Care Hospitals (n = 4822)
Number of hospital beds, median (IQR^c^)	501 (336 to 740)	489 (380 to 652)	101 (36 to 227)
Number of ICU^d^ beds, median (IQR)	64 (42 to 95)	54 (39 to 76)	8 (2 to 22)
Region, n (%)			
Northeast	37 (25)	109 (24)	600 (12)
Midwest	41 (28)	100 (22)	1423 (30)
South	44 (30)	175 (39)	1853 (38)
West	26 (18)	63 (14)	946 (20)
Urbancity, n (%)			
Division (>2.5 million persons)	40 (27)	131 (29)	687 (14)
Metropolitan (50K^e^–2.5 million)	106 (72)	309 (69)	2068 (43)
Micropolitan (10K–50K)	2 (1)	5 (1)	872 (18)
Rural (<10K)	0	2 (1)	1195 (24)
Teaching status, n (%)			
Large teaching	73 (49)	148 (33)	355 (7)
Small teaching	34 (23)	141 (32)	910 (19)
Non-teaching	41 (28)	158 (35)	3557 (74)
Financial status, n (%)			
Nonprofit	112 (76)	356 (80)	2745 (57)
For profit	11 (7)	41 (9)	784 (16)
Government	25 (17)	50 (11)	1293 (27)

a *Hospitals reporting extracorporeal membrane oxygenation cases to the Extracorporeal Life Support Organization or extracorporeal membrane oxygenation procedure codes in Medicare discharge claims*;

b *hospitals reporting more than 315 annual claims for medical mechanical ventilation*,

c *interquartile range*,

d *intensive care unit*,

e *thousand*.

### Direct High Capability Severe Acute Respiratory Failure Center Geographic Access

**Figure 1:**
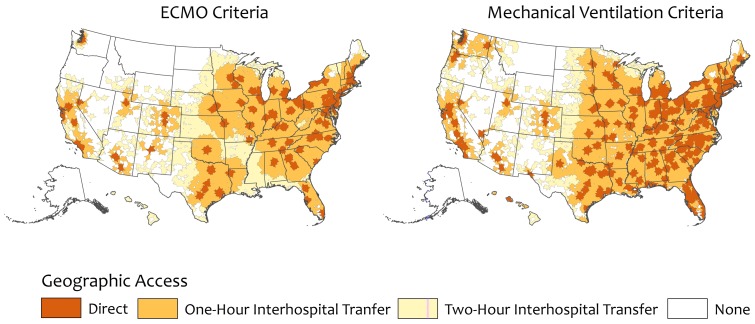
Continuous Albers equal area projections showing geographic access to high capability severe acute respiratory failure centers in the United States. Geographic access is defined using two separate hospital criteria: provision of ECMO or high annual volume of mechanical ventilation. The dark orange areas show regions with direct high capability severe acute respiratory failure center geographic access. Medium orange areas show regions with geographic access after a one-hour ground or air interhospital transfer. Light orange areas show regions with geographic access after a two-hour ground or air interhospital transfer. *ECMO: extracorporeal membrane oxygenation; MV: high annual volume of mechanical ventilation.*

**Figure 2:**
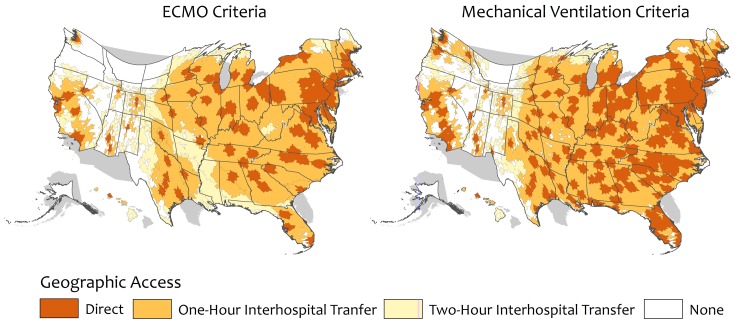
Gastner-Newman transformations of continuous Albers equal area projections of the United States showing geographic access to high capability severe acute respiratory failure centers in the United States. Geographic access is defined using two separate hospital criteria: provision of ECMO or high annual volume of mechanical ventilation. The dark orange areas show regions with direct high capability severe acute respiratory failure center geographic access. Medium orange areas show regions with geographic access after a one-hour ground or air interhospital transfer. Light orange areas show regions with geographic access after a two-hour ground or air interhospital transfer. *ECMO: extracorporeal membrane oxygenation; MV: high annual volume of mechanical ventilation.*

Direct high capability center geographic access was 58.5% for the ECMO criteria and 79.0% for the high volume mechanical ventilation criteria (Table 2). Regionally, direct high capability center geographic access ranged from 47.9% in the South for ECMO to 92.2% in the Northeast for high volume mechanical ventilation. Nine states had no direct geographic access to high capability centers using the ECMO criteria and three states by high mechanical ventilation criteria (Figures 1 & 2).

**Table 2:** Geographic Access to High Capability Severe Acute Respiratory Failure Center in the United States.

	ECMO Critiera^a^			High Volume Mechanical Ventilation Criteria^b^		
	Direct	One-Hour Interhospital Transfer	Two-Hour Interhospital Transfer	Direct	One-Hour Interhospital Transfer	Two-Hour Interhospital Transfer
United States, %	58.5	87.5	96.4	79.0	96.5	98.6
Northeast, %	79.4	98.7	99.8	92.2	99.9	99.9
Connecticut	85.9	99.9	99.9	99.6	100	100
Maine	45.1	90.4	98.4	61.7	96.1	99.1
Massachusetts	81.7	99.8	99.8	95.9	99.9	99.9
New Hampshire	69.7	100	100	52.7	100	100
New Jersey	85.7	99.8	99.8	99.5	100	100
New York	78.2	98.8	99.8	91.9	99.9	99.9
Pennsylvania	84.1	100	100	92.0	100	100
Rhode Island	63.0	99.8	99.8	99.8	99.9	99.9
Vermont	0	45.7	100	61.0	100	100
Midwest, %	57.7	92.8	98.7	73.4	97.1	99.3
Illinois	75.9	100	100	80.1	100	100
Indiana	50.6	100	100	70.8	100	100
Iowa	18.9	99.1	100	42.6	100	100
Kansas	31.8	61.6	100	64.3	94.0	100
Michigan	65.1	93.2	99.5	83.0	96.1	99.5
Minnesota	63.7	89.0	99.0	73.8	97.7	99.4
Missouri	52.7	90.9	99.3	70.4	99.3	99.3
Nebraska	60.3	85.9	99.4	60.3	86.2	99.4
North Dakota	0	0	37.2	22.7	47.8	85.8
Ohio	59.6	100	100	89.5	100	100
South Dakota	0	7.1	68.9	28.2	60.2	75
Wisconsin	56.2	97.7	99.7	52.6	95.2	99.7
South, %	47.9	85.0	99.6	78.6	99.2	99.8
Alabama	23.7	79.5	99.9	71.6	100	100
Arkansas	0.4	20.8	99.6	39.4	99.6	99.6
Delaware	92.2	100	100	71.8	100	100
District of Columbia	100	100	100	100	100	100
Florida	47.2	88.0	99.4	93.7	99.6	99.8
Georgia	60.0	98.0	99.8	77.5	99.9	99.9
Kentucky	43.9	99.9	100	69.8	100	100
Louisiana	9.5	32.4	98.9	60.4	99.4	99.4
Maryland	87.2	99.8	99.8	89.5	99.9	99.9
Mississippi	0	16.6	99.9	47.9	99.9	99.9
North Carolina	41.1	99.7	99.8	84.0	99.8	99.8
Oklahoma	34.2	92.3	100	61.8	99.2	100
South Carolina	38.6	99.9	99.9	81.9	99.9	99.9
Tennessee	49.9	83.7	100	80.6	100	100
Texas	63.0	82.4	99.0	77.4	96.9	99.6
Virginia	52.8	99.8	99.9	86.5	99.9	99.9
West Virginia	7.1	90.2	99.9	53.3	99.9	99.9
West, %	59.4	77.6	86.6	74.3	88.8	95.1
Alaska	0	0	0	0	0	0
Arizona	77.3	87.4	97.0	78.4	91.9	97
California	71.7	97.2	99.5	85.3	98.8	99.5
Colorado	70.9	90.5	98.0	72.2	90.3	98
Hawaii	68.7	69.6	69.6	69.1	85.1	97.7
Idaho	0	1.8	47.0	8.2	22.1	89.2
Montana	0	0	0	0	1.9	36.9
Nevada	19.5	22.9	96.5	89.6	94.2	96.5
New Mexico	42.4	57.9	94.1	8.3	16.1	51.3
Oregon	0	0	8.8	60.2	80.5	97.9
Utah	71.6	89.8	96.7	74.0	94.8	97.9
Washington	49.5	62.4	68.8	74.6	95.7	98.8
Wyoming	0	26.7	65.7	0	26.7	65.7

a *Hospitals reporting extracorporeal membrane oxygenation cases to the Extracorporeal Life Support Organization or extracorporeal membrane oxygenation procedure codes reported in Medicare discharge claims*;

b *hospitals reporting more than 315 annual claims for medical mechanical ventilation*.

### Indirect High Capability Severe Acute Respiratory Failure Center Geographic Access through Interhospital Transport

A total of 274 high capability centers reported helipads to ADAMS. Nationally, 87.5 and 96.5% of the adult population had geographic access to a high capability center with one-hour interhospital transport using ECMO and high volume mechanical ventilation criteria, respectively (Table 2). Geographic access increased to 96.4% and 98.6% with two-hour interhospital transport, respectively. High capability center geographic access varied by state and region, with Western states having the lowest regional and state-level geographic access (Figures 1 & 2).

## Discussion

Geographic access to high capability severe acute respiratory failure centers varies substantially across states and regions in the United States. An estimated 58.5 to 79.0% of the population has direct geographic access to a high capability center. Geographic access increases to 96.4 to 98.6% when accounting for interhospital transport of up to two hours. This suggests the existing hospital infrastructure is geographically capable of reaching most Americans who develop severe acute respiratory failure; however, some states had no high capability centers, and many rural areas were without timely access using more restrictive interhospital transport assumptions.

Our analysis provides important preliminary insight into geographic access to high capability centers for severe acute respiratory failure in the United States. We defined geographic access using accessibility, which is the relationship between the location of patients and the location of health care resources. This is an important component of health care access [35]; however, true access to these centers involves more than just accessibility. Practically, access also requires complex coordination efforts across multiple hospitals and explicit regional planning to address other key access domains. These domains include capacity (e.g., the relationship between demand and ICU supply–including ICU beds and ICU personnel), accommodation (e.g., the relationship between the development of acute respiratory failure and the ability of the health care system to move the patient to a high capability center), affordability (e.g., the relationship between the cost of care and the patient's or insurer's ability to pay), and acceptability (e.g., the patient's or hospital's comfort with characteristics of client-provider relationship) [36]. All of these domains require further study as we attempt to organize the health system to best meet the needs of patients with severe acute respiratory failure.

The final domain, acceptability, includes both the hospital perspective and patient choice. While there may be survival benefits to moving the location of ICU care hundreds of miles to a regional center, a patient-centered approach optimally incorporates patient and family preferences. Our model defined transports up to two hours by helicopter to reach a high capability center as “available” – corresponding to approximately two hundred forty miles. A moderate additional travel burden for regional center care may be acceptable to some patients [37]; however, there is likely a threshold for others, beyond which they would prefer local hospital services [38]. The success of regionalized care will certainly depend on finding a balance between system capability and patient preferences.

We acknowledge that the criteria we used to identify high capability severe acute respiratory failure centers were proxies, and other elements of care are likely associated with high quality, beyond ECMO and high volumes. Other interventions associated with improved quality include the ability to use prone positioning [4], use of paralytic agents [5] or routinely applying low tidal volumes in ARDS [7]. However, in the absence of a consensus definition, the provision of ECMO or high annual volumes of mechanical ventilation may identify hospitals with these other capabilities. Indeed, it is notable that there are currently no standardized definitions for categorizing critical care resources. Such definitions are urgently needed to facilitate further evaluation of regional critical care organization. We believe the criteria we examined are well supported by the available literature and thus have face validity for identifying high quality severe acute respiratory failure care.

In evaluating the validity of our definition, it is worth noting that high mechanical ventilation volume and ECMO identified 498 high capability centers in the United States, which is similar to the number of level 1 or 2 trauma system hospitals (n = 445) [21]. The annual number of severe ARDS cases is lower than the estimated 678,000 severely injured patients treated at trauma centers each year [39], though ICUs do not exclusively provide ARDS care. Similarly, in the United States there are 925 primary stroke centers [40] and as of 2008 there were 298 hospitals performing coronary artery bypass graft surgery [41]. This indicates that our criteria identified a similar proportion of centers to other formally and informally regionalized care systems.

Geographic access to hospitals meeting the ECMO criteria ranged form 58.5 to 96.4%, depending on interhospital transport assumptions. Quantifying geographic access to state and regional ECMO-capable hospitals is especially important for pandemic planning, as a subset of patients with severe acute respiratory failure may not improve with conventional ventilator support. ECMO infrastructure expansion will not be practical in all areas, therefore hospitals in geographic “white spaces” should consider developing regional transfer agreements in anticipation of these events as well as for the routine care of the most severely ill patients with acute respiratory failure.

Further study is required to measure other aspects of resource availability, including regional center staffing and measures of ICU capacity [42]. As stressed by the United States Department of Health and Human Services' Hospital Preparedness Program, regional capability should be viewed as a community attribute, rather than a facility one [43].

Our analysis has other limitations. We did not address redundant geographic access to high capability centers. Several regions had tightly clustered hospitals meeting our center criteria, raising the question if similar geographic access would be possible with fewer high capability centers. Furthermore, structure is not equivalent to organization. We specified transfer relationships between referring and receiving hospitals that may not occur in practice. Additionally, high capability centers may already be operating near or at capacity, making them unable to serve as referral centers. Coordination of regional critical care for severe acute respiratory failure will clearly require planning and protocols to achieve efficient and high quality regional care.

Our ECMO center criteria also had limitations. We identified patients who received ECMO through Medicare claims, rather than an all-payer database. We may have missed potential ECMO centers because they provide ECMO only to young patients or exclusively serve a Medicare Advantage population (who do not appear in fee-for-service Medicare claims). Further, the ELSO criterion only identified adult hospitals performing veno-venous ECMO. Finally, we did not include pediatric hospitals, though some may perform adult ECMO cannulation in nearby adult hospital centers.

Our modeling approach also included real-world simplifications. For example, we did not account for the effects of extreme winter weather or daily traffic patterns. Additionally, we used the geometric centroid of each census block group to calculate the catchment area population. As such, our estimates should be considered in a “best case scenario” context, and may overestimate true geographic access.

Despite these limitations, our findings have practical significance. First, we identified several states have no geographic access to high capability centers. These states should immediately consider the capabilities of their hospitals to transfer patients with severe acute respiratory failure to the closest high capability center. Second, we identified many states with incomplete geographic access. Further work is needed to evaluate the cost of infrastructure expansion against competing regionalization priorities, recognizing that geographic access to high capability severe acute respiratory failure centers may be implausible.

Our analysis provides a conceptual framework for evaluating intensive care infrastructure. Action is needed to focus attention on other domains of access by engaging stakeholders in a discussion of regionalized critical care. Important next steps include standardizing criteria for ICU levels of care, creating protocols for transferring patients with severe acute respiratory failure to high capability centers and developing regional systems of audit and feedback to promote a continuous improvement process. The alignment of health care systems with public health efforts has the potential to improve the routine care of patients with severe acute respiratory failure, as well as to improve health system resilience during times of additional strain.

## Conclusions

Geographic access to high capability severe acute respiratory failure centers varied substantially across states and regions in the United States, depending on center criteria. An estimated 96.4% and 98.6% of the national population had geographic access to a high capability center; however, this degree of access required substantial interhospital transfer of many patients, including up to two hours by rotatory air transport. Adequate referral center access in the case of disasters and pandemics will depend highly on local and regional care coordination across political boundaries.
